# Rétinoblastome bilatéral: à propos d'un cas

**DOI:** 10.11604/pamj.2014.17.141.4027

**Published:** 2014-02-27

**Authors:** Hakima Elouarradi, Rajae Daoudi

**Affiliations:** 1 Université Mohammed V Souissi, Service d'Ophtalmologie A de l'Hôpital des Spécialités, Centre Hospitalier Universitaire, Rabat, Maroc

**Keywords:** Rétinoblastome, tumeur, mutation génétique, Retinoblastoma, tumor, genetic mutation

## Image en medicine

**Figure 1:**
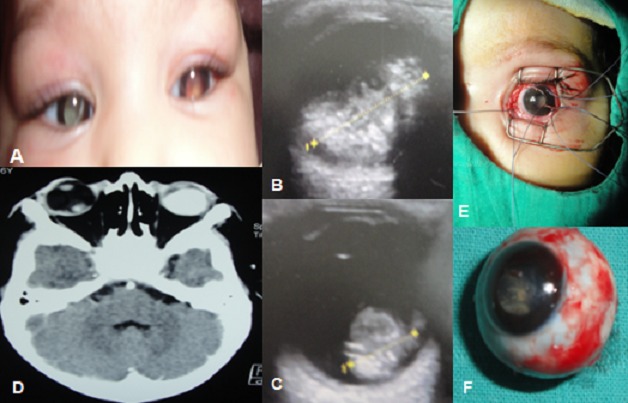
A) Leucocorie bilatérale; (B,C) Aspect échographique montrant des tumeurs hyperéchogènes par rapport au vitré de taille plus grande au niveau de l’œil gauche (B) par rapport à l’œil droit droit (C); D) Aspect scannographique montrant un rétinoblastome bilatéral; E) Aspect peropératoire; F) L’œil gauche énuclée

Nourrisson de 6 mois, issu d'une grossesse bien suivie, menée à terme, avec notion de consanguinité parentale du 2 ème degré et sans cas similaires dans la famille, unique de ses parents, emmené par ses parents aux urgences pour leucocorie bilatérale (A). Le nourrisson a bénéficié d'un examen ophtalmologique sous anesthésie générale, un examen général pédiatrique et un bilan radiologique. L'examen ophtalmologique note un segment antérieur normal au niveau des 2 yeux, avec un cristallin clair en ODG. Le fond d'oeil retrouve au niveau de l'oeil droit des petites masses rétiniennes blanchâtres, et au niveau de l'oeil gauche volumineuse tumeur blanchâtre vascularisées avec des flocons blanchâtres intra vitréens. L’échographie oculaire (B,C) et la tomodensitométrie orbito-cérébrale (Figure 1D) objectivent un aspect en faveur d'un rétinoblastome bilatéral. Le bilan d'extension clinique et paraclinique est normal. Après évaluation multidisciplinaire onco- ophtalmologique et accord signé des parents, le nourrisson a bénéficié d'une chimiothérapie néoadjuvante, puis d'une énucléation de l'oeil gauche (E,F) avec mise en place d'une bille, en prévoyant un traitement conservateur de l'oeil droit. Avec une surveillance très rapprochée et à long terme. En rappelant les parents de la nécessité d'un examen ophtalmologique de dépistage chez leurs futurs enfants. Le Rétinoblastome fait partie des maladies dites “orphelines”. C'est la tumeur intraoculaire maligne la plus fréquente chez l'enfant mettant en jeu le pronostic visuel et vital, d'origine génétique (mutation du gène RB 13q14). Il est bilatéral dans 40% des cas. Les deux symptômes les plus fréquents révélateurs sont la leucocorie et le strabisme.

